# Editorial to the Special Issue “Theoretical and Computational Polymer Science: Physics, Chemistry, and Biology”

**DOI:** 10.3390/polym17162242

**Published:** 2025-08-19

**Authors:** Hector Eduardo Roman

**Affiliations:** Department of Physics, University of Milano-Bicocca, Piazza della Scienza 3, 20126 Milano, Italy; hector.roman@unimib.it

This Editorial provides a concise review of the contributions featured in this Special Issue (SI), which is dedicated to the theoretical aspects of polymers in physics, chemistry, and biology, covering both their structural and dynamical properties. The SI was originally conceived to highlight algorithms for generating suitable chain configurations in diverse environments, including disordered structures, fractals, and confined biological complexes such as proteins and the cell nucleus. The research articles collected and presented here address recent challenges of both general and specific interest across these three scientific domains, with extensions into engineering applications. The Special Issue also features a review article focusing on the fundamental properties and characterization of linear polymers, their modeling and scaling behavior, and their embedding in deterministic and disordered fractals. We expect readers will benefit from this collection of works, which spans a broad spectrum of current research and aims to provide a unified perspective on the physical and chemical properties of these complex and remarkable systems.

The SI brings together experts working across different fields of polymer science and technology, and comprises 13 research contributions organized into three main categories: physics [1,2,3,4,5,6], chemistry [7,8,9,10,11], and biology [12,13]. Contribution [14] concludes this SI. In the following, we briefly summarize each contribution separately.

Block Copolymers and Brushes: Self-consistent field theory (SCFT) is a powerful theoretical framework for studying many-body systems, where complex interactions are handled using a mean-field approximation. In contribution [1], the authors present a simplified 3D SCFT algorithm that employs real-space methods with adaptive discretization, enhancing both the accuracy and efficiency of numerical computations. This algorithm is applied to the study of polymeric material surfaces and is tested on two distinctly different systems: block copolymer films and polymer brushes. A key innovation is the use of finer contour discretization at the grafted chain ends, which improves spatial resolution in regions affected by external forces. This approach results in one of the most accurate SCFT implementations for 3D polymeric systems to date.

Material Models for Limb Orthoses: Fused deposition modeling is widely used in the biomedical sector due to its versatility in processing various materials. However, selecting the most suitable material for a specific application can be challenging. In particular, understanding the mechanical behavior of polymeric materials is crucial to achieving the desired performance. In contribution [2], the authors use a finite element numerical approach to analyze three material models: the Bergstrom–Boyce, the three-network, and the three-network viscoplastic models. These models are employed for studying the mechanical behavior of three commonly used polymers: acrylonitrile butadiene styrene (ABS), polylactic acid (PLA), and polyethylene terephthalate glycol (PETG), in the context of personalized upper limb orthoses. This approach integrates theoretical modeling with experimental validation to assess the mechanical suitability of these materials for producing accurate, customized orthotic devices.

Hydrogen Desorption in Polymer Materials: In contribution [3], the authors numerically investigate the diffusion of hydrogen through a polymer material in storage containers of various geometries, including cylindrical, spherical, and sheet-shaped forms. They quantify the results using the desorption equilibrium time—defined as the time at which hydrogen diffusion loss reaches saturation. This equilibrium time exhibits an exponential increase with the square of both the thickness and diameter for cylindrical specimens. For spherical containers, it is found to be proportional to the square of the diameter, and for sheet-shaped containers, to the square of the thickness. The model calculations, based on solutions to the diffusion equation, show good agreement with experimental data, offering a reliable tool for predicting the time-dependent behavior of hydrogen desorption in polymer materials.

Carbon Fiber Fabric-Reinforced Polyetheretherketone: In modern industrial applications, thermoplastic resins are predominantly selected for their high-temperature resistance and robust performance. Among these, polyetheretherketone (PEEK) stands out as a semi-crystalline, aromatic, and high-performance engineering polymer. PEEK is widely recognized for its exceptional thermal stability, radiation and corrosion resistance, superior dimensional stability, and excellent electrical properties. Combined with its outstanding processability, these features have earned PEEK a reputation as one of the most advanced thermoplastic materials in current use. In [4], the authors focus on carbon fiber fabric–reinforced PEEK (CFF/PEEK) composites. In their study, PEEK resin samples were first subjected to a saturated hygroscopic treatment at 70 °C. Tensile and compressive tests were then performed on the composites both before and after moisture exposure, generating mechanical property data under different environmental conditions. The work examines the hygroscopic behavior of pure PEEK resin and CFF/PEEK composites, and it compares the mechanical performance, failure modes, and fracture morphologies of the samples prior to and following hygroscopic treatment. The findings provide valuable insights into how hygrothermal environments affect the mechanical behavior of CFF/PEEK composites. Such knowledge is crucial for guiding the design and engineering applications of thermoplastic composite materials, particularly in fields requiring long-term durability under variable environmental conditions.

Mesoscopic Phase Separation in 3D gels: Contribution [5] reports molecular dynamics (MD) simulations of mesoscopic phase separation in three-dimensional gels using a bead–spring Lennard–Jones model. The study focuses on the formation of high-density phase (HDP) networks, driven by the interplay of short-range attractions and long-range elastic forces. HDP network morphology is highly sensitive to temperature, spring stiffness, and particle density; higher stiffness yields faceted structures, elevated temperatures produce wider but less dense networks, and higher densities generate more compact morphologies. Network evolution proceeds through initial HDP precipitation followed by surface minimization. These insights are relevant for applications where the gel microstructure is critical, such as ion-gel solid electrolytes and the mechanical behavior of polymeric gels.

Carbon Fiber/Glass Fiber (CF/GF) Hybrid-Reinforced Resin Matrix: A flywheel energy storage system stores and releases energy by converting electrical energy to mechanical energy through the acceleration and deceleration of a rotating rotor. The radial stability of the rotor can be significantly enhanced by using carbon fiber/glass fiber (CF/GF) hybrid-reinforced resin matrix composites as the rotor material. The inclusion of glass fiber not only provides a smooth transition between the metal hub and the composite rim but also reduces material costs, addressing the economic limitations of rotors made solely from carbon fiber-reinforced composites. In contribution [6], the authors employ finite element analysis to study a three-dimensional model of such a composite rotor, focusing on the behavior of the metal hub and the hybrid composite rim. The radial stress distribution during operation is used to optimize the CF/GF mixing ratio. A high-speed rotation scenario at 18,000 rpm is simulated to evaluate the rotor’s safety and reliability. Based on these results, the material selection and structural design of the rotor are refined to avoid resonance regions, demonstrating that the flywheel can safely operate at high rotational speeds.

Swelling Response of Nitrile Butadiene Rubber (NBR): In contribution [7], the authors perform an equilibrium swelling test to evaluate the swelling behavior of NBR with varying acrylonitrile content. A predictive model for the oil resistance of NBR was developed using the three-dimensional Hansen solubility parameter (HSP) and the modified Flory–Huggins interaction parameter (χHSP). Results show that the energy difference (Ra) between NBR and the solvent, derived from HSP values, correlates with swelling behavior in an inverse S-shaped trend, with significant swelling occurring at Ra < 8 MPa^1/2^. For model development, the χHSP parameter was calculated and correlated with the swelling response using both exponential and logarithmic fittings, yielding two predictive models that incorporate all relevant influencing factors. These models accurately estimate the swelling and oil resistance of NBR-based components in biofuels, demonstrated here with biodiesel and IRM 903 test oil. By identifying and avoiding high-swelling regions, the approach provides a reliable prediction of oil resistance for NBR seals, hoses, and gaskets in emerging fuel environments.

Cross-linked Polyethylene (XLPE): XLPE is widely used in high-voltage direct-current (HVDC) cable insulation, traditionally produced via dicumyl peroxide (DCP) cross-linking. However, byproducts from the DCP process can increase the electrical conductivity of the insulation, prompting interest in byproduct-free cross-linking methods. In contribution [8], the authors discuss a promising alternative involving in situ cross-linking between ethylene–glycidyl methacrylate copolymer and 1,5-disubstituted pentane via reactive compounding. Ab initio quantum chemical calculations, based on density functional theory (DFT), were performed for 18 reaction pathways, revealing that epoxy groups react with the disubstituted pentane to form a covalently linked XLPE network through epoxy ring-opening and ester formation, with the pentane acting as the cross-linker. Among the functional groups examined, carboxyl groups were the most reactive, and a pathway denoted as RTS1 was kinetically favored with a Gibbs energy barrier of 1.95 eV. This byproduct-free XLPE cross-linking approach provides a viable alternative to DCP, paving the way for thermoplastic insulation materials with improved electrical performance for next-generation HVDC power cables.

Dynamical Properties of Polyurea (PUR): PUR has gained widespread use as a protective coating due to its excellent mechanical durability and energy absorption properties. In contribution [9], using material characterization techniques and MD simulations, the authors compare PURs synthesized with different macrodiol structural units to better understand the relationship between microstructure and energy absorption. The findings reveal that PURs based on polycarbonate diols exhibit high tensile strength, superior toughness, and favorable energy dissipation behavior, as demonstrated through stress–strain curves, glass transition analysis, phase imaging, and dynamic storage/loss modulus measurements. MD simulations further showed that external energy from shear deformation is primarily absorbed via non-bond interactions, particularly influenced by fractional free volume, interaction energy, total energy variations, and hydrogen bond dynamics. Crucially, hydrogen bonding between soft and hard segments plays a dominant role in controlling the mechanical and dynamic responses of PURs. The close agreement between experimental characterization and MD predictions highlights the potential of molecular-level insights to guide the structural design of PURs with optimized energy absorption performance.

New Graftable Antioxidant for Improved XLPE Insulation: In conventional XLPE insulation, the presence of additives—such as antioxidants and cross-linking agents—can significantly increase electrical conductivity. Consequently, reducing antioxidant content is a promising strategy to further minimize conductivity. Contribution [10] introduces the dual-functional antioxidant 5-allyloxy-2-hydroxyl-3-tert-butylbenzophenone (5ATB), specifically designed to enhance insulation performance. The antioxidant behavior and UV-induced grafting reaction of 5ATB were investigated using DFT. Calculations reveal that the Gibbs energy barrier for the reaction of 5ATB with O_2_ is approximately 0.5 eV lower than that of a polyethylene chain with O_2_, confirming its superior antioxidative potential. Natural bond orbital analysis further identifies the hydrogen-accepting site of 5ATB on the CH_2_ group of the C=C double bond. This mechanistic insight highlights a novel approach to antioxidant design, offering a pathway for developing next-generation low-conductivity XLPE insulation materials for advanced power cable applications.

Dielectric Permittivity of Polymer Nanocomposites: In contribution [11], a revised Poon–Shin (PS) model incorporating interphase effects is proposed to predict the dielectric permittivity of polymer nanocomposites reinforced with spherical nanoparticles. In this case, each nanoparticle and its surrounding interphase are treated as a core–shell equivalent particle, allowing the composite to be modeled as a homogeneous mixture of the polymer matrix and equivalent particles. The dielectric calculation proceeds in two steps: first, the permittivity of the core–shell particle is determined; then, the overall composite permittivity is evaluated using the modified PS framework. Model predictions show strong agreement with experimental data when interphase properties are accurately incorporated. The study also examines how nanoparticle, interphase, and matrix characteristics influence dielectric behavior. This enhanced PS model provides a useful theoretical basis for designing polymer–nanoparticle composites with optimized dielectric performance for advanced electronics and energy storage applications.

Model Polymers vs. Globular Proteins: In contribution [12], the behavior of linear chain molecules is explored through Monte Carlo (MC) simulations of standard polymer chains represented as tethered spheres, evaluated across both low- and high-temperature regimes. The simulated polymer chains are then systematically compared with the three-dimensional structures of globular proteins, using experimental data retrieved from the Protein Data Bank (PDB). The study provides a detailed structural analysis encompassing both local features—such as bond connectivity and short-range packing—and non-local features, including long-range folding patterns and contact maps. By examining the distribution of closest contacts and applying symmetry-based considerations, the authors attempt to reconcile the seemingly different conformational behaviors of idealized model chains and real protein structures. This approach not only deepens the understanding of polymer–protein structural analogies but also offers insights into how chain connectivity, packing, and symmetry govern the emergence of three-dimensional conformations in complex biomolecular systems.

Polyelectrolyte Adsorption: Contribution [13] investigates the adsorption behavior of polyelectrolyte (PE) chains onto spherical particles with heterogeneously charged surfaces. The primary focus is on identifying critical adsorption conditions and understanding the influence of a low-dielectric particle core. Using Metropolis Monte Carlo simulations within the canonical ensemble, the PE chains are allowed to explore the configurational space around the binding particle. Remarkably, two distinct adsorption–desorption transitions are identified when the particle carries a net surface charge of the same sign as the PE, leading to a non-monotonic dependence of the critical surface charge density required for electrostatically driven adsorption. The study reveals an enhanced adsorption affinity for particles with heterogeneous charge distributions, particularly when the particle has a low dielectric constant. This contrasts with the weaker adsorption observed for homogeneously charged, low-dielectric particles. The increased affinity arises when the Debye screening length in the surrounding medium is comparable to the size of oppositely charged surface patches, facilitating localized electrostatic attraction. The Discussion section highlights several real-world applications of such PE–particle systems, especially in the context of complex formation between polyelectrolytes and globular proteins that exhibit dipolar or patchy surface charge distributions. Examples include biologically relevant proteins such as insulin and bovine serum albumin (BSA), where the findings may help explain binding mechanisms critical to drug delivery, biosensing, and protein–polymer interactions.

Review: Finally, contribution [14] offers an overview of recent applications of linear polymers and polymer networks across condensed matter physics, chemistry, and biology by briefly discussing a selection of papers published between 2022 and 2024. It is structured into three main subsections: physics (focusing on coarse-grained model simulations and the structural properties of materials), chemistry (covering quantum mechanical calculations, environmental considerations, and rheological behavior of viscoelastic composites), and biology (addressing macromolecules, proteins, and biomedical applications). The central part of the work reviews theoretical aspects of linear polymers, with a particular emphasis on self-avoiding walk (SAW) chains on regular lattices as well as on deterministic and random fractal structures. The latter are modeled as critical percolation clusters, with the concept of multifractality, typical of such complex systems, being explored in detail. A detailed discussion of the reptation model and its broad range of applications is included. The topics of protein folding and protein evolution are also addressed, with key challenges and open questions highlighted. An experimental section introduces the most relevant physical aspects of linear polymers pertinent to the review. The final two sections are devoted to applications: one in materials science, focusing on fractal characteristics of plasma-treated polymer surfaces and the growth of polymer thin films, and the other in biology, which considers, among other topics, long linear polymers like DNA confined within finite domains.

Among the different issues reviewed in [14], the following themes were considered: the structure of linear polymers in 3D lattices [15], polymer melts [16], elastic and plastic responses of these glassy materials [17], bottlebrushes [18], amphiphilic copolymers such as block polyelectrolytes [19], surfactants at water–oil interfaces [20], polymeric crystals [21], nucleation of hard spheres [22], fibrous materials [23,24], anomalous dynamics and mixing behavior [25,26], translocation through a nanomembrane [27], polymerization of hexanediol dimethacrylate (HDDMA) [28], relaxation of nitrile buttadiene rubber [29], polymeric biocomposites [30,31], insulating aramid/SiO_2_ mixtures [32], charge polymers [33], bioplastics and their accumulation in the environment [34,35], polymerization of branched polymers [36], synthetic hydrogels [37], block copolymers [38], covalently functionalized graphene and carbon nanotubes [39], an artificial network of neurons [40], polymer/graphene interfaces [41], translocation of polymers through narrow channels [42,43], porous polyimide [44,45], additive manufacturing [46,47,48,49], conjugated polymers [50,51], block copolymers and brushes [52,53], plasma techniques for the treatment of polymeric surfaces [54,55,56], polymer electrolyte for fuel cells [57,58,59], nitrile butadiene rubber [60], dendrimers [61,62,63,64], pyrolysis of polystyrene [65,66], oilfield scale inhibitors [67], insulating materials [68,69], gas desorption of H_2_ from polymer media [70,71,72], biodegradable polymer materials [73,74,75,76], gas separation membranes [77,78,79], polymer electrolytes for Li-based batteries [80,81,82,83], epoxy resins [84,85,86,87], viscoelastic behavior of polymer fluids [88,89], natural fiber-reinforced polymer composites [90], elongational rheology [91,92], polymer rheology theories [93,94], orientational relaxation [95,96,97], large-scale molecular simulations [98,99,100], actin filaments [101,102,103], conformational dynamics of GLP-2 peptide [104,105,106], soft vesicle–polymer chain conformations [107,108], entangled polymer networks [109,110,111], polyelectrolyte pore translocation [112,113,114,115], protein adsorption dynamics [116,117,118,119], biomedical applications of polymers [120,121,122], and polymer hydrophilic networks for energy storage [123,124].
